# A Diagnostic Dilemma: A Case of Neurosarcoidosis Without Systemic Sarcoidosis

**DOI:** 10.7759/cureus.42844

**Published:** 2023-08-02

**Authors:** Ephrem Sedeta, Nosakhare P Ilerhunmwuwa, Rachna Hindu Pahlani, Henry Aiwuyo, Mustafa Wasifuddin, Ifeanyi Uche, Narek Hakobyan, Jamal Perry, Sima Terebelo

**Affiliations:** 1 Medicine, Brookdale University Hospital Medical Center, Brooklyn, USA

**Keywords:** intracranial mass, meningeal mass, seizure, intracranial sarcoidosis, neurosarcoidosis

## Abstract

Sarcoidosis is a multisystem granulomatous disorder of unknown etiology characterized by non-caseating granulomas in involved organs. Approximately 10% of patients with sarcoidosis exhibit central nervous system involvement. However, the occurrence of isolated neurosarcoidosis without concurrent systemic signs is very rare, affecting less than 1% of patients. We report a case of isolated neurosarcoidosis in a previously healthy patient who initially presented with a single episode of seizure and loss of consciousness. Brain MRI showed T2/fluid-attenuated inversion recovery (FLAIR) hyperintense extra-axial soft tissue mass over the left cerebral convexity measuring approximately 14 mm in maximum depth. Excisional biopsy of the brain mass showed chronic non-caseating granulomatous inflammation with epitheloid cells that was consistent with sarcoidosis. Treatment with high dose-steroids led to significant clinical improvement. At a two-year follow-up, there were no signs of systemic disease or recurrence of the meningeal mass. This case emphasizes the rarity of such presentation, diagnostic difficulties, and the importance of high suspicion and timely management to prevent debilitating neurologic complications.

## Introduction

Sarcoidosis is a multisystem granulomatous disorder of unknown etiology characterized by non-caseating granulomas in involved organs [1]. The lungs are affected in nearly 90% of patients and account for most of the associated morbidity and mortality [2]. Other commonly involved tissues include the skin (up to 30%), eyes (10-25%), and mediastinal and hilar lymph nodes (90%) [2]. Neurologic complications occur in about 5-10% of patients with sarcoidosis and symptoms range from meningitis with cranial nerve involvement to seizures or extremity paralysis [1,3]. While neurologic complaints in patients with known sarcoidosis raise the possibility of neurosarcoidosis, it is very uncommon for sarcoidosis to solely affect the central nervous system (CNS) without concurrent systemic signs. Approximately 10% of patients with sarcoidosis exhibit central nervous system involvement; however, the occurrence of isolated neurosarcoidosis without concurrent systemic signs is very rare, affecting less than 1% of patients [4,5]. The diagnostic process for intracranial neurosarcoidosis poses challenges due to its non-specific clinical presentation, including symptoms such as headache, seizures, signs of meningeal irritation, cranial neuropathy, or extremity paralysis [6,7]. Neuroradiological imaging also presents non-specific characteristics, such as enhancing parenchymal mass lesions, leptomeningeal involvement, hypothalamus and pituitary involvement, cranial nerve involvement, hydrocephalus, or dural involvement, further making the diagnosis challenging [8]. Early diagnosis in these rare cases remains of paramount importance to prevent disabling complications.

## Case presentation

A previously healthy 27-year-old male patient of African American descent presented to the emergency department after experiencing a single seizure episode. The patient had no significant past medical history, including seizures or other neurological disorders. Family members witnessed the seizure, describing it as generalized shaking of the entire body and upward rolling of the eyes, which lasted 1-2 minutes, followed by a brief loss of consciousness. There was no tongue biting or bowel or bladder incontinence. The patient was reportedly confused and drowsy after the seizure subsided. The patient denied nausea, vomiting, headache, neck stiffness, or sensitivity to light. He denied smoking cigarettes but drank alcohol socially, and used marijuana occasionally. Upon conducting a comprehensive neurological examination, including a thorough evaluation of gait, strength, reflexes, sensory examination, presence of pyramidal signs, cerebellar signs, cranial nerves, and mental status examination, no abnormalities were detected.

**Figure 1:**
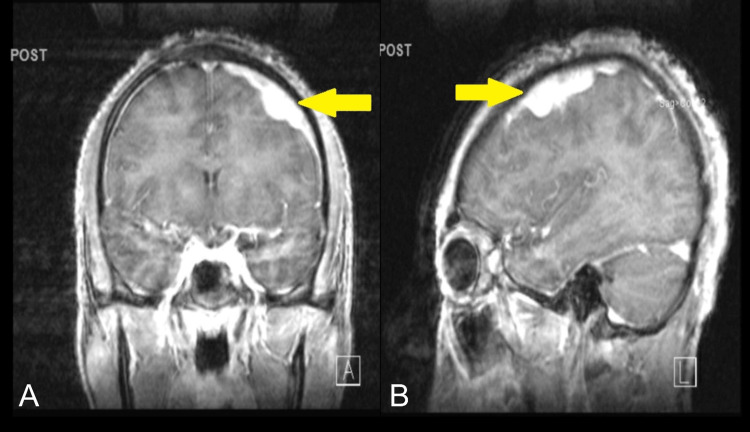
Brain MRI image of extra-axial meningeal mass. The images show (A) extensive abnormal extra-axial soft tissue density measuring 14 mm in maximum depth over the left cerebral convexity in post-contrast coronal T1-weighted brain MRI image and (B) sagittal view of the T1-weighted brain MRI.

The results of complete blood count, ESR, serum electrolytes (including calcium, phosphorus, and magnesium), and tests of liver and renal function were within normal reference range. The HIV test was negative, and rapid plasma reagin and QuantiFERON tests were done to assess for potential prior infections of syphilis and tuberculosis. Brain MRI showed T2/FLAIR hyperintense extra-axial soft tissue mass over the left cerebral convexity measuring approximately 14 mm in maximum depth (Figures 1A, 1B).

**Figure 2:**
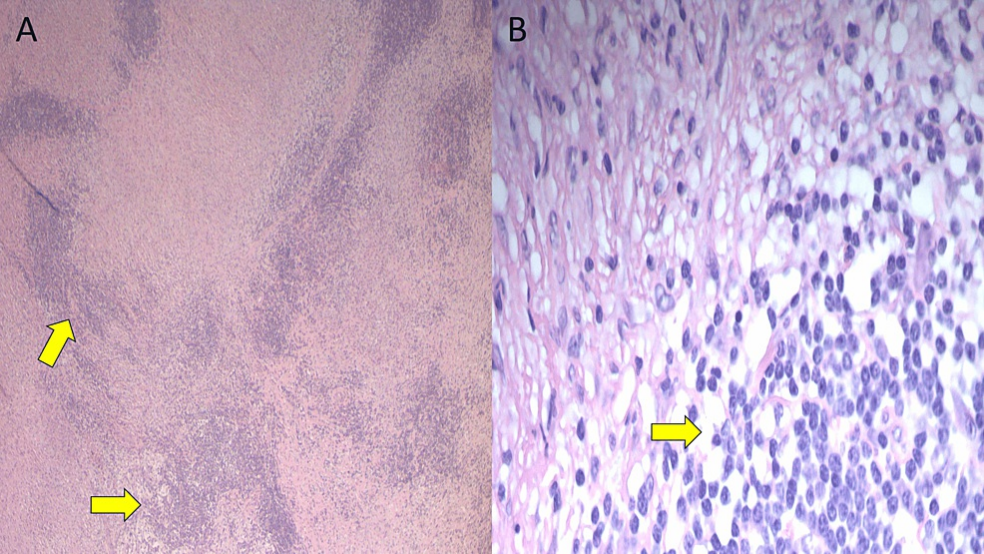
Histological section of meningeal mass. (A) H&E slide of meninges showing extensive chronic inflammation with granulomatous features and (B) H&E slide showing chronic meningeal inflammation with palisading histiocytic reaction.

**Figure 3:**
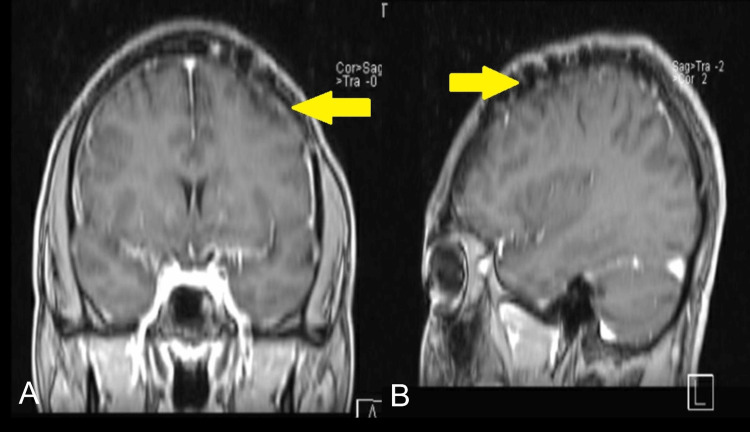
Brain MRI Image at one-year follow-up. (A) Post-contrast coronal T1-weighted brain MRI image at one-year follow-up and (B) post-contrast sagittal T1-weighted brain MRI image at one-year follow-up.

Based on brain CT and MRI findings, a brain biopsy was performed. The pathology report of the biopsy showed chronic non-caseating granulomatous inflammation with epithelioid cells that were consistent with sarcoidosis (Figures 2A, 2B). No organisms were seen on acid-fast, Grocott's methenamine silver, or mucicarmine special stains. The patient was initially treated with prednisone 40 mg daily and later switched to azathioprine 50 mg daily as a steroid-sparing agent. The patient is currently on azathioprine 150 mg daily. He has developed focal seizures with occasional left upper extremity jerky movements for which he is on levetiracetam 1500 mg twice a day and lacosamide 50 mg twice a day. He is regularly being monitored for drug adverse effects. He has follow-ups at our rheumatology and neurology clinics and continues to follow-up. So far, there are no signs of systemic disease or recurrence of the meningeal mass (Figures 3A, 3B).

## Discussion

Sarcoidosis is a systemic disease with a heterogenous clinical presentation, primarily affecting young adults. Although its incidence varies among geographical regions and genetic backgrounds, it is more common among women and individuals of African descent in North America [9]. The occurrence of isolated neurosarcoidosis without concurrent systemic signs is very rare, affecting less than 1% of patients [6]. Isolated neurologic sarcoidosis remains a diagnostic challenge [10,11].

Neurosarcoidosis exhibits diverse clinical presentations, including parenchymal, extra-axial, and leptomeningeal lesions, with meningeal involvement of the skull base, hypothalamus, and pituitary gland being the most frequently affected sites [6]. In neurosarcoidosis, virtually any neurologic symptom may arise in isolation because sarcoidosis can affect any part of the nervous system [7]. The most common clinical manifestation of neurosarcoidosis is cranial neuropathy with unilateral or bilateral facial nerve involvement [1,7]. The optic nerve is the second most commonly affected cranial nerve [1,7]. Other sarcoidosis-related neurologic complications include aseptic meningitis (10-20%), seizure (5-10%), neuropsychiatric conditions (20%), neuroendocrine dysfunctions (10-15%) [10], and peripheral neuropathies (15%) [1,12]. Less commonly, isolated neurosarcoidosis can also present as isolated or multiple enhancing intracranial parenchymal masses and might be mistaken for a primary or secondary tumor of the central nervous system, as in this case [4]. Clinical symptoms can manifest suddenly or gradually, and they can also advance chronically over time [13].

The accurate diagnosis of neurosarcoidosis requires a stepwise approach to identify characteristic clinical presentations and tissue biopsy findings that demonstrate non-caseating granulomas and exclude other multisystem granulomatous diseases [14,15]. Cerebrospinal fluid analysis can establish the presence of intrathecal inflammation and rule out infectious and neoplastic processes. Frequent CSF abnormalities that provide important evidence for active inflammation include elevated total protein, mononuclear cell pleocytosis, low glucose, occasionally elevated CSF angiotensin-converting enzyme (ACE) level, elevated IgG index, and oligoclonal bands [16]. The Neurosarcoidosis Consortium Consensus Group has proposed diagnostic criteria for neurosarcoidosis based on clinical presentation and utilizing various diagnostic evaluations [17].

The differential diagnosis of granulomatous meningeal lesions includes sarcoidosis, tuberculosis, parasitic and fungal infections, granulomatosis with polyangiitis (GPA), rheumatoid arthritis, and hypertrophic pachymeningitis [18]. In our patient, since all special organism stains were negative, tuberculosis, mycoses, or other rare infections would be very unlikely. Moreover, infectious granulomas are commonly caseating, in contrast to our patient who had non-caseating granulomas. Granulomatosis with polyangiitis (Wegener’s granulomatosis) is a necrotizing vasculitis that often presents with non-specific constitutional symptoms and characteristic lesions involving the sinuses, lungs, and kidneys. It can rarely present with a meningeal mass; however, the biopsy would show necrotizing granulomas with necrotizing vasculitis [18]. Anti-neutrophil cytoplasmic antibodies are commonly present as well which was not the case in our patient. Rheumatoid arthritis was unlikely, as our patient did not have the characteristic clinical findings. Hypertrophic pachymeningitis is a fibrosing inflammatory disorder marked by localized or diffuse thickening of the dura mater and often caseating granulomas which were not consistent with our patient’s findings.

As sarcoidosis is a multiorgan disease, patients with suspected or confirmed neurosarcoidosis require a careful initial evaluation for systemic disease. The chest x-ray and computed tomographic scan of the chest performed on this patient did not show the presence of hilar adenopathy or pulmonary parenchymal changes typically associated with pulmonary sarcoidosis. The abdominal ultrasound did not reveal liver parenchymal changes. Serum calcium and vitamin D levels were also within normal limits and serum ACE assay was not elevated.

Due to the severe morbidity linked with neurosarcoidosis and the potentially harmful consequences of relapsing/progressive disease, the majority of patients undergo initial treatment with corticosteroids [19]. Afterward, they are shifted to immunosuppressive therapy as a means of long-term management. Although there is a lack of large randomized, double-blind, placebo-controlled treatment trials for neurosarcoidosis, the available evidence from small active-controlled trials and case series supports the approach of ongoing immunosuppression for better long-term outcomes in treated patients [20,21]. The initial immunosuppressive treatment of neurosarcoidosis is common with either methotrexate, azathioprine, or mycophenolate mofetil [22]. In our patient’s case, due to elevated transaminases and concern for liver involvement, we elected to start azathioprine as it has lower liver toxicity and is commonly used in autoimmune liver disease. In the event of treatment failure, other second and third-line therapies such as adalimumab and infliximab (tumor necrosis factor-alpha {TNF-α} antagonists) as well as rituximab can be considered [23,24]. In specific clinical scenarios, symptomatic therapies such as hormone replacement treatment for pituitary dysfunction, seizure management, and neurosurgical interventions for hydrocephalus or intracranial mass effect may be necessary [6]. Historically, nearly 10% of patients die as a direct result of the inflammatory process or its treatment; however, current treatment strategies have substantially improved patient survival [19].

## Conclusions

This is an uncommon case of isolated neurosarcoidosis that was the first presentation of sarcoidosis in a previously healthy patient. The diagnosis of isolated neurosarcoidosis requires a strong clinical suspicion as tissue biopsy of the CNS lesions may not always be possible. Awareness of the different presentations of neurosarcoidosis is crucial for early recognition and to prevent disabling complications.
